# Comprehensive Chemical and Biological Evaluation of *Rosa damascena* Plant Parts and Industrial Residues Using FTIR‐ATR and LC–MS/MS

**DOI:** 10.1002/fsn3.72072

**Published:** 2026-07-02

**Authors:** Burak Bayrak, Hafize Yuca, Yasemin Beyza Budak, Satuk Buğra Alkuyruk, Elif Beyza Özer, Gülnur Ekşi Bona, Bilge Aydın, Mehmet Karadayı, Yusuf Gülşahin, Alptuğ Atila, Gamze Göger, Songul Karakaya

**Affiliations:** ^1^ Department of Analytical Chemistry, Faculty of Pharmacy Ataturk University Erzurum Türkiye; ^2^ Department of Pharmacognosy, Faculty of Pharmacy Ataturk University Erzurum Türkiye; ^3^ Department of Pharmaceutical Microbiology, Faculty of Pharmacy Ataturk University Erzurum Türkiye; ^4^ Graduate School of Health Sciences Ankara University Ankara Türkiye; ^5^ Department of Pharmacognosy, Faculty of Pharmacy Ondokuz Mayıs University Samsun Türkiye; ^6^ Department of Pharmaceutical Botany, Faculty of Pharmacy Istanbul University‐Cerrahpaşa İstanbul Türkiye; ^7^ Department of Pharmacognosy, Faculty of Pharmacy Erzincan Binali Yıldırım University Erzincan Türkiye; ^8^ Department of Biology, Faculty of Science Atatürk University Erzurum Türkiye; ^9^ Department of Pharmacognosy, Faculty of Pharmacy Afyokarahisar Health Sciences University Afyon Türkiye; ^10^ Department of Pharmaceutical Botany, Faculty of Pharmacy Ataturk University Erzurum Türkiye

**Keywords:** antioxidant activity, biomass valorization, enzyme inhibition, genotoxicity assessment, phytochemical profiling, *Rosa damascena*

## Abstract

This study comprehensively evaluated the phytochemical composition, biological activities, and biosafety profile of different organs and processing by‐products of *Rosa damascena*, with particular emphasis on valorizing pruning residues and distillation wastes. Extraction yields varied among plant parts, with leaves (29.69%) and flowers (14.15%) providing higher recoveries than buds (7.98%) and branches (7.91%). FTIR‐ATR spectroscopy differentiated lignocellulosic woody tissues from phenolic‐ and ester‐rich floral materials and confirmed the characteristic aliphatic profile of rose oil. Biological activity assays demonstrated strong *α*‐glucosidase inhibition, particularly in flower extracts (IC_50_: 37 μg/mL), surpassing acarbose, while branches, buds, and leaves also exhibited high activity; in contrast, hydrosol and essential oil showed no measurable inhibition. Antioxidant capacity assays revealed notable radical scavenging and reducing activities, especially in buds, branches, and flowers, whereas hydrosol and essential oil displayed limited effects. Moderate antimicrobial activity was observed mainly against *Staphylococcus aureus* and *Candida albicans* (MIC: 625 μg/mL). LC–MS/MS analysis identified 20 phenolic compounds, with quinic acid as the predominant constituent in buds and flowers. In addition, significant levels of gallic and ellagic acids were detected in distillation wastewater, highlighting the bioactive potential of processing residues. Ames/*Salmonella, E. coli* WP2, and *Allium cepa* assays confirmed the absence of genotoxic effects at tested concentrations. Overall, the findings demonstrate that *R. damascena* organs and industrial by‐products represent valuable sources of bioactive compounds, exhibiting in vitro *α*‐glucosidase and *α*‐amylase inhibitory activities associated with potential antidiabetic effects, as well as notable antioxidant capacity, thereby supporting their prospective utilization in functional food and nutraceutical applications.

## Graphical abstract

*Rosa damascena* flowers, buds, leaves, branches, and hydrodistillation by‐products were investigated using FTIR‐ATR, LC–MS/MS, and a series of biological assays. The integrated approach highlighted the chemical diversity and bioactive potential of both plant materials and industrial processing residues, supporting their sustainable valorization.

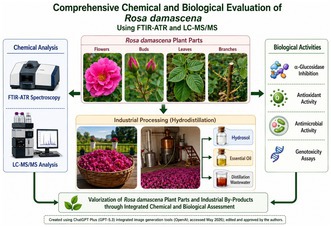

## 1 Introduction

Diabetes mellitus (DM) is a chronic metabolic disorder caused by insulin deficiency or resistance, characterized by hyperglycemia in fasting or postprandial states. Individuals with DM face a higher risk of severe health complications, leading to increased healthcare costs, reduced quality of life, and higher mortality rates. Rapid urbanization and sedentary lifestyles have significantly accelerated the prevalence of DM worldwide. In 2009, an estimated 150 million people were affected, with projections reaching 300 million by 2025. Alarmingly, nearly half of DM cases remain undiagnosed for up to a decade, suggesting its true global prevalence may be much higher (Cho et al. 2018; Gholamhoseinian and Fallah 2009; Kaul et al. 2013). Synthetic inhibitors like acarbose and voglibose have been developed to inhibit *α*‐glucosidase and *α*‐amylase enzymes, but they often cause side effects such as bloating, digestive issues, and liver dysfunction. As a result, natural inhibitors with fewer side effects are preferred. Numerous in vitro and in vivo studies have explored the antidiabetic potential of such phytochemicals (Alsalti et al. 2022).

Alzheimer's disease (AD) is the leading cause of dementia, impacting over 24 million people worldwide, primarily older adults. Its global prevalence is expected to double within the next two decades (Adeghate et al. 2013).

DM increases the risk of vascular dementia and AD, influenced by age, lifestyle, and environmental factors. Insulin resistance and metabolic syndrome may contribute to cognitive decline, while genetic and environmental interactions shape this link. Recent research suggests antidiabetic medications may reduce AD‐related brain plaques (Plastino et al. 2010).

Antioxidants counteract free radicals, which are linked to aging and diseases like cancer, multiple sclerosis, Parkinson's, dementia, autoimmune disorders, and asbestosis. To mitigate the harmful effects of free radicals, the body relies on antioxidants. However, free radical damage remains a concern, highlighting the need to explore natural products for their antioxidant potential (Achuthan et al. 2003; Liu et al. 2020; Mawarni et al. 2020).

The *Rosa* L. genus is known as one of the most popular ornamental plants with its pleasant smell and beautiful appearance and belongs to the Rosoideae subfamily of the Rosaceae family. The rose plant is one of the most cultivated ornamental plants in the world today. The history of the rose is largely accepted to originate from Western Asia and partly from Europe. It is widely seen in European and Middle Eastern countries, especially Iran, Afghanistan and Turkey. It is also grown in Bulgaria, Russia, Egypt, France, India and Morocco (Torusdağ and Bakkalbaşı 2022). *Rosa* encompasses over 200 species and more than 18,000 varieties. Among these, *Rosa damascena* stands out as one of the most significant ornamental plants of the Rosaceae family. Renowned for its premium aromatic oil, this species is extensively cultivated and finds prominent applications in the perfumery and pharmaceutical industries (Kendir and Köroğlu 2021). *R. damascena*, whose homeland is Iran, has a natural distribution area in the Northern Hemisphere and is grown especially in Türkiye and Bulgaria, as well as France, Lebanon, India, Russia, China, Morocco, Mexico, Italy and other European countries. *R. damascena* is a very thorny and pink‐flowered plant formed by hybridization of *R. gallica* L. and *R. phoenicia* Boiss. species (Ağar 2018).

It is an erect, perennial, deciduous shrub that can grow up to 2 m. The leaves are imparipinnate and consist of 5–7 leaflets. The flower bud is oblong, the sepals bend backwards after the flowers open; the tube is long, usually widening at the top. It has many flowers of a deep pink color. The flowers consist of 5 sepals and numerous petals, stamens, and pistils. The sepals are lanceolate and green, the petals are deep pink and broadly ovate (Akram et al. 2020; Kendir and Köroğlu 2021).

In Traditional Iranian Medicine, *Rosa damascena* flower decoctions have been valued for treating chest and abdominal pain, menstrual disorders, and digestive issues, acting as a mild laxative and cardiotonic to strengthen the heart. Rose water has traditionally been used as an antiseptic for eye washing and an oral disinfectant. Rose petals, cooked with sugar or honey, were employed to cool the body and mind (Mahboubi 2016). Native American tribes used root decoctions of *R. damascena* to ease children's coughs, while rose oil steam therapy was beneficial for allergies, headaches, and migraines. In Indian Ayurvedic medicine, rosebuds are regarded as mental tonics, heart‐friendly, and astringent. Dried buds have been used as a laxative and flavoring agent (Boskabady et al. 2011; Singh et al. 2023).

Various parts of *R. damascena* are traditionally used for multiple purposes, and its extracts demonstrate significant pharmacological activities, including hypnotic, analgesic, antispasmodic, laxative, antidiabetic, antimicrobial, anti‐HIV, anti‐inflammatory, and antioxidant effects (Singh et al. 2023).

Türkiye and Bulgaria are the world's leading producers of rose oil, each contributing approximately 1.5 tons annually to the global production, which totals around 3 tons per year. In Turkey, rose oil production is predominantly concentrated in the regions of Isparta, Burdur, and Afyon, with increasing attention toward organic cultivation (Baser and Arslan 2014).

Recent studies have extensively investigated the phytochemical composition of *R. damascena*, revealing that different plant parts contain distinct classes of bioactive compounds. Flowers are particularly rich in volatile constituents (e.g., citronellol, geraniol, and their esters) and phenolic compounds, while leaves and branches are characterized by lignocellulosic structures and varying levels of phenolic acids and flavonoids. Buds represent an intermediate stage with developing secondary metabolites, whereas essential oil is dominated by non‐polar volatile components. In contrast, hydrosol and distillation wastewater contain more polar compounds, including phenolics such as gallic and ellagic acids. In recent years, increasing attention has been given to the valorization of rose oil processing residues, with studies demonstrating their significant antioxidant, antimicrobial, and functional food potential (Kolev et al. 2025; Dinkova et al. 2022; Trendafilova et al. 2023).

Despite the extensive use of *R. damascena* in perfumery, cosmetics, and traditional medicine, existing studies have primarily focused on its essential oil and floral extracts, with limited attention given to other plant parts and industrial by‐products (Nayebi et al. 2017). However, *R. damascena* processing generates significant amounts of by‐products such as hydrosol and distillation residues, which may still contain valuable bioactive compounds (Lebkiri et al. 2026). Although the chemical composition and biological properties of essential oil have been widely investigated, including antimicrobial and antioxidant effects, studies integrating multiple plant organs and processing residues within a single analytical framework remain scarce (Verešová et al. 2024). Furthermore, variations in chemical composition across plant parts and environmental conditions have been reported, yet comparative evaluations linking phytochemical composition with biological activity across different matrices are still limited (Uçar et al. 2024). Therefore, a comprehensive and integrative approach is needed to evaluate both plant organs and industrial by‐products of *R. damascena*, in order to better understand their bioactive potential and support sustainable biomass valorization.

To better address this gap, the present study adopts an integrative analytical approach combining FTIR‐ATR, LC–MS/MS, bioactivity assays, and biosafety evaluation within a single framework. FTIR‐ATR provides rapid and non‐destructive characterization of functional groups, enabling the differentiation of major chemical classes across plant matrices, while LC–MS/MS allows precise identification and quantification of individual phenolic constituents. When coupled with biological activity assays (e.g., enzyme inhibition and antioxidant capacity), these techniques enable the direct association of chemical composition with functional properties. In addition, the inclusion of biosafety assessment (genotoxicity assays) ensures that the biological potential of the samples is evaluated alongside their safety profile. Such a comprehensive approach is still limited in the literature for *R. damascena*, particularly in studies simultaneously addressing multiple plant organs and industrial by‐products. Therefore, integrating phytochemical characterization with bioactivity and safety evaluation provides a more complete understanding of the valorization potential of *R. damascena* biomass.

In this context, the present study aimed to comprehensively evaluate the phytochemical composition, biological activities, and biosafety profile of different organs of R. damascena (flowers, buds, leaves, and branches), together with industrial processing by‐products, including hydrosol, essential oil, and distillation wastewater. The study specifically focuses on (i) characterizing compositional differences among plant parts and processed materials using FTIR‐ATR and LC–MS/MS analyses, (ii) assessing their antioxidant capacities and enzyme inhibitory activities (*α*‐glucosidase, *α*‐amylase, and cholinesterases), (iii) evaluating antimicrobial effects, and (iv) determining genotoxic safety using Ames/*Salmonella, E. coli* WP2, and *Allium cepa* assays. By integrating compositional analysis with bioactivity and safety evaluation, this research contributes to the sustainable valorization of rose biomass and its industrial by‐products as potential functional food and nutraceutical resources.

## 2 Material and Method

### 2.1 Plant Material

The representatives of AVOS ORGANICS, Mr. Pharmacist Allahverdi KARACA and Mr. Pharmacist Volkan ZÜLALOĞLU, cultivate *Rosa damascena* Mill. (*Qızıl Gül*) in Iğdır, where they produce rose oil and rose water. They have supported our project by providing various products and byproducts related to rose cultivation and processing. The products supplied by the company include:

- Flowers.
- Buds.
- Essential oil (rose oil).
- Hydrosol (rose water).
- Branches (pruning residues).
- Leaves (pruning residues).
- Wastewater remaining in the boiler after distillation.

From the plant materials, 50 g of dried samples were obtained for each part, while 500 mL of liquid products and 1 mL of essential oil were provided.

### 2.2 Extraction

The dried plant materials provided by the company were individually ground into powder. For each sample, 50 g of powdered material was extracted with methanol. Methanol was selected as the extraction solvent due to its high efficiency in extracting a broad range of polar and moderately polar phytochemicals, particularly phenolic compounds, which are known to be associated with biological activities such as antioxidant and enzyme inhibitory effects.

Prior to extraction, the samples were macerated in methanol overnight to facilitate solvent penetration and improve extraction efficiency. The extraction was then carried out in a water bath at 40°C, a temperature chosen to enhance mass transfer while minimizing thermal degradation of heat‐sensitive compounds. The mixtures were filtered at 8‐h intervals, and the obtained filtrates were collected and combined. This sequential extraction approach was applied to ensure exhaustive recovery of extractable constituents.

After completion of the extraction process, all combined filtrates were subjected to rotary evaporation at 40°C under reduced pressure to remove methanol completely and obtain dry extracts. The resulting concentrates were transferred from round‐bottom flasks into small containers and further kept in a fume hood for approximately 3 days to ensure complete removal of any residual solvent. The dried extracts obtained from R. damascena—flower, bud, branch (pruning residue), and leaf (pruning residue)—were then used for subsequent activity assays.

Although alternative extraction systems such as aqueous or green solvents (e.g., ethanol or water‐based systems) are commonly used, methanol was preferred in this study to ensure maximum recovery and comparability of phenolic constituents across different plant matrices under standardized laboratory conditions.

Rose water (hydrosol), rose oil (essential oil), and the wastewater remaining in the boiler after distillation were supplied directly by the company and were used without any further treatment in the activity assays.

### 2.3 Fourier Transform Infrared Spectroscopy‐Attenuated Total Reflectance (FTIR‐ATR) Analysis

FTIR–ATR spectra of *Rosa damascena* extracts were recorded using an IRSpirit FTIR Spectrophotometer (Shimadzu Corporation, Kyoto, Japan) equipped with a QATR‐S single‐reflection ATR sampling accessory. The measurements were carried out within the spectral range of 4000–400 cm^−1^ at a resolution of 4 cm^−1^. For each sample, 20 scans were accumulated to improve the signal‐to‐noise ratio.

Prior to each measurement, the ATR crystal surface was carefully cleaned with ethanol and dried to avoid cross‐contamination. A background spectrum was recorded before each sample analysis under identical instrumental conditions and automatically subtracted from the sample spectrum. Liquid samples were directly placed onto the ATR crystal, while dried extract samples were finely ground and pressed onto the crystal surface to ensure proper contact. All measurements were performed at room temperature. Each sample was analyzed in triplicate to ensure reproducibility, and the spectra presented in this study represent the average of these three independent measurements. Raw spectral data were imported into RStudio for preprocessing and further analysis. Baseline correction was performed using the Asymmetric Least Squares (ALS) method to improve spectral quality and minimize background drift. Characteristic absorption bands were identified according to their wavenumbers (cm^−1^) and interpreted based on literature data to determine the functional groups present in the samples. The “ggplot2”, “baseline”, “reshape2” and “fmsb” packages from the Rstudio program were used for data preprocessing and visualization (Civaş et al. 2026).

### 2.4 *α*‐Glucosidase Inhibitory Activity Assay

The *α*‐glucosidase inhibitory activity was determined using a modified version of the method described by Bachhawat et al. (2011). In a 96‐well microplate, 50 μL of phosphate buffer (50 mM, pH 6.9), 10 μL of *α*‐glucosidase enzyme (1 Unit/mL), and 20 μL of plant extracts at concentrations ranging from 1 to 5000 μg/mL were mixed and incubated at 37°C for 5 min. Subsequently, 20 μL of 3 mM p‐nitrophenyl‐α‐D‐glucopyranoside (pNPG) was added as a substrate, and the mixture was incubated at 37°C for 30 min. The reaction was terminated by adding 50 μL of 0.1 M sodium carbonate. All solutions were prepared in a buffer system. Acarbose was used as a positive control. The amount of yellow‐colored p‐nitrophenol (pNP) formed was measured at 405 nm. Each assay was performed in triplicate. The percentage inhibition was calculated using the following formula (Karakaya et al. 2023):

$$\text{Inhibition}\;\left(\%\right)=\left(1-\Delta A_{40\text{5sample}}/\Delta A_{40\text{5control}}\right)\times 100$$

### 2.5 *α*‐Amylase Inhibitory Activity Assay

The *α*‐amylase inhibitory activity was determined following the method of Nampoothiri et al. (2011). Equal volumes (100 μL) of the sample (concentration range: 1–5000 μg/mL) and a 1% starch solution prepared in 20 mM sodium phosphate buffer (containing 6 mM sodium chloride, pH 6.9) were incubated in a microplate at 25°C for 10 min. After incubation, 100 μL of porcine pancreatic *α*‐amylase (0.5 mg/mL) was added to each well, and the samples were further incubated at 25°C for 10 min. The reaction was terminated by adding 200 μL of dinitrosalicylic acid (DNS) color reagent, followed by incubation at 100°C for 5 min. The samples were then cooled to room temperature, and 50 μL of each reaction mixture was transferred to a 96‐well microplate. To each well, 200 μL of distilled water was added to dilute the reaction mixture, and the absorbance was measured at 540 nm. Results were compared with the control, and acarbose was used as a positive control. The percentage inhibition was calculated using the following formula (Karakaya et al. 2023):

$$\text{Inhibition}\;\left(\%\right)=\left(1-\Delta A_{54\text{0sample}}/\Delta A_{54\text{0control}}\right)\times 100$$

### 2.6 Acetylcholinesterase (AChE) and Butyrylcholinesterase (BChE) Inhibitory Activities

The AChE and BChE enzyme inhibition assays were performed with minor modifications to the method described by Ingkaninan et al. (2000).

In a 96‐well microplate, the following components were mixed:

- 125 μL of 5,5′‐dithiobis‐(2‐nitrobenzoic acid) (3 mM, DTNB, Ellman's Reagent),
- 25 μL of substrate (15 mM; acetylthiocholine iodide for AChE and butyrylthiocholine iodide for BChE),
- 50 μL of Tris–HCl buffer (50 mM, pH 8), and
- 25 μL of the sample solution.

Subsequently, 25 μL of the enzyme (AChE or BChE) was added to the mixture. The reaction mixture was incubated for 10 min for AChE inhibition and 15 min for BChE inhibition. Absorbance was measured spectrophotometrically at 405 nm. Donepezil was used as the positive control. Each experiment was performed in triplicate. The percentage of enzyme inhibition was calculated using the following formula (Karakaya et al. 2023):

$$\text{Inhibition}\;\left(\%\right)=\left(1-\Delta A_{40\text{5sample}}/\Delta A_{40\text{5control}}\right)\times 100$$

### 2.7 2,2‐Diphenyl‐1‐Picrylhydrazyl (DPPH) Radical Scavenging Capacity Assay

The DPPH^•^ radical scavenging capacities of *Rosa damascena* extracts were determined based on the method developed by Blois (1958) with slight modification made by Aydın et al. (2023). In this method, *α*‐tocopherol and trolox were used as standards, and their antioxidant effects were tested using a 1 mM DPPH^•^ solution. The percentage inhibition values of the standards at specific concentrations (1–100 μg/mL) against DPPH^•^ were evaluated to determine the concentration range for the samples. The correlation coefficients (*r* values) of the calibration curves created based on the percentage inhibition values of the standard substances at the prepared concentrations were determined as 0.99. Serial dilutions of the samples were prepared within the concentration range of 10–100 μg/mL, and their antioxidant activities were assessed against 1 mM DPPH^•^ solution. All measurements were recorded at 517 nm against a blank consisting of 99% ethanol. Each assay was performed in triplicate. The DPPH^•^ radical scavenging capacity of the extracts was calculated using the following formula:

$$\%\text{Inhibition}=\left[\left(A_{\text{control}}-A_{\text{Sample}}\right)/A_{\text{control}}\right]\times 100$$

*A*: Absorbance value at 517 nm.

### 2.8 2,2′‐Azino‐Bis(3‐Ethylbenzothiazoline‐6‐Sulfonic Acid) (ABTS) Radical Scavenging Capacity Assay

The ABTS^•+^ radical scavenging capacities of *Rosa damascena* extracts were determined based on the method developed by Re et al. (1999) with slight modification made by Aydın et al. (2023). In this method, *α*‐tocopherol and trolox were used as standards, and their antioxidant effects were tested using a 2 mM ABTS^•+^solution. The percentage inhibition values of the standards at specific concentrations (1–100 μg/mL) against ABTS^•+^ were evaluated to determine the concentration range for the samples. The correlation coefficients (*r* values) of the calibration curves created based on the percentage inhibition values of the standard substances at the prepared concentrations were determined as 0.99. Serial dilutions of the samples were prepared within the concentration range of 10–100 μg/mL, and their antioxidant activities were assessed against the 2 mM ABTS^•+^ solution. All measurements were recorded at 734 nm against a blank containing phosphate buffer. Each assay was performed in triplicate. ABTS^•+^ radical scavenging capacity of the extracts was calculated using the following formula: ABTS^•+^ scavenging capacity was calculated according to the following equation:

$$\%\text{Inhibition}=\left[\left(A_{\text{control}}-A_{\text{Sample}}\right)/A_{\text{control}}\right]\times 100$$

*A*: Absorbance value at 734 nm.

### 2.9 Cupric Reducing Antioxidant Capacity (CUPRAC) Assay

The copper‐reducing antioxidant capacities of *Rosa damascena* extracts were determined by making minor adjustments to the method developed by Apak et al. (2004). In this method, *α*‐tocopherol and trolox were used as standards. The CUPRAC reagent was prepared by mixing 10 mM CuCl_2_, 7.5 mM neocuproine, and 1 M ammonium acetate buffer (pH 7.0). The antioxidant effects of the standards were tested within the concentration range of 1–100 μg/mL to establish calibration curves. The correlation coefficients (*r* values) of the calibration curves created based on the absorbance values of the standard substances at the prepared concentrations were determined as 0.99. Serial dilutions of the samples were prepared within the concentration range of 10–100 μg/mL, and their antioxidant activities were assessed using the CUPRAC reagent mixture. After incubation at room temperature for 30 min, the absorbance values were recorded at 450 nm against a reagent blank. Each assay was performed in triplicate. The antioxidant capacities of the extracts were calculated from the calibration curves of the standard substances and expressed as μg trolox equivalent per mL extract.

### 2.10 Ferric Reducing Antioxidant Power (FRAP) Assay

The ferric‐reducing antioxidant powers of *Rosa damascena* extracts were determined by making minor adjustments to the method developed by Benzie and Strain (1996). In this method, *α*‐tocopherol and trolox were used as standards. The FRAP reagent was freshly prepared by mixing 300 mM acetate buffer (pH 3.6), 10 mM TPTZ solution in 40 mM HCl, and 20 mM FeCl_3_·6H_2_O in a ratio of 10:1:1 (v/v/v). The antioxidant effects of the standards at specific concentrations (1–100 μg/mL) were evaluated to determine the concentration range for the samples. The correlation coefficients (*r* values) of the calibration curves created based on the absorbance values of the standard substances at the prepared concentrations were determined as 0.99. Serial dilutions of the samples were prepared within the concentration range of 10–100 μg/mL, and their antioxidant activities were assessed using the FRAP reagent. After incubation at 37°C for 30 min, the absorbance values were recorded at 593 nm against a reagent blank. Each assay was performed in triplicate. The antioxidant power of the extracts was calculated from the calibration curves of the standard substances and expressed as μg trolox equivalent per mL extract.

### 2.11 Determination of Antimicrobial Activity

The antimicrobial potential of each test substance was evaluated against the bacterial strains *Staphylococcus aureus* ATCC 6538, *Pseudomonas aeruginosa* ATCC 9027, and the yeast *Candida albicans* ATCC 10231 using the microdilution method (Gulluce et al. 2007). Fresh liquid cultures of the test microorganisms were prepared in appropriate growth media obtained from the culture collection of the Department of Biology, Faculty of Science, Atatürk University. Ciprofloxacin (MedChemExpress, USA), ampicillin (Tokyo Chemical Industry, Japan), and fluconazole (Deva Pharmaceutical Company) were used as standard antimicrobial agents. Mueller Hinton Agar (MHA), Mueller Hinton Broth (MHB), and Tryptic Soy Agar (TSA) were supplied by Biolife Italiana (Italy). The concentrations of antibiotics (0.25–128 μg/mL) and extracts (78.125–5000 μg/mL) were prepared using water and 10% dimethyl sulfoxide (DMSO).

The Clinical and Laboratory Standards Institute (CLSI 2006, 2008) procedures for aerobic bacteria (M100‐S16) and yeasts (M27‐A) were followed with minor modifications to determine the minimum inhibitory concentration (MIC) values (CLSI 2006, 2008; Göger et al. 2018).

The strains were inoculated onto MHA plates and incubated at 37°C for 24 h. Single colonies were then transferred into tubes containing MHB and incubated again for 24 h at 37°C. Following incubation, the cultures were adjusted to McFarland 0.5 standard (approximately 10^8^ CFU/mL) using a densitometer. Two‐fold serial dilutions of extracts and antimicrobial agents were prepared. Subsequently, 50 μL of microorganism suspension was inoculated into each well. After incubation at 35°C–37°C for 16–20 h, 15 μL of resazurin reagent (0.1 mg/mL) was added to evaluate microbial growth. The plates were further incubated for 1–2 h at 37°C, and MIC values were determined at the end of the incubation period.

### 2.12 Biosafety Assessment

The Ames/*Salmonella* and *E. coli* WP2 bacterial reversion assays and the *Allium* test were used to assess the biosafety potential of the *R. damascena* extracts and the essential oil.

The Ames/*Salmonella* assay was performed according to a well‐documented protocol previously described by Mortelmans and Zeiger (2000). *S. tyhimurium* TA1535, TA1537, TA97a, TA98 and TA100 were chosen as the tester strains. The known mutagens sodium azide (NaN_3_), 9‐aminoacridine (9‐AA) and 4‐nitro‐*o*‐phenylenediamine (4‐NPD) were used as positive controls and dimethyl sulfoxide (DMSO) was the negative/vehicle control. To determine applicable test concentrations of the extracts and the essential oil, the viability assay was performed that confirmed normal growth of the background lawn, lack of reduction in cell survival and acceptable spontaneous revertant colony numbers. In the mutagenicity assay, 50 μL of the test material solution and 100 μL of the bacterial culture (approx. 10^8^ bacterial cell) were added into 500 μL of sodium phosphate bufer (0.1 mM, pH 7.4). Then, it was gently mixed with 2 mL of molten top agar supplemented with biotin and trace amount of histidine. The mixture immediately poured on to glucose minimal (GM) agar plates. The cultures were incubated at 37°C for 72 h, and the histidine‐independent revertant colonies were scored after the incubation. The plate incorporation method was used to evaluate genotoxicity results. The results are expressed as mean ± standard deviation (SD), calculated from pooled data across three independent experiments, each conducted with two replicate plates per concentration (total *n* = 6). Mutagenic index (M_IND_) was calculated according to the following equation:

$$M_{\mathrm{IND}}=R_{\text{Test}}/R_{\text{Negative}}$$

*R*_Test_: Average number of revertants in the test plate. *R*_Negative_: Average number of revertants in the negative/vehicle control.

A test material was determined as genotoxic when M_IND_ was greater than 2 and a dose‐dependent relationship was observed.

The *E. coli* WP2 assay, a complementary test for the Ames/*Salmonella* assay, was performed according to the procedure previously described by Mortelmans and Riccio (2000). *Escherichia coli* WP2*uvrA* was chosen as the tester strain. N‐Methyl‐N′‐nitro‐N‐nitrosoguanidine was the positive control and dimethyl sulfoxide (DMSO) was used as the negative/vehicle control. All experimental steps of the viability and mutagenicity tests were the same as the Ames/*Salmonella* assay detailed above. The only procedural difference was the addition of a trace amount of tryptophan (0.05 mM) instead of biotin and histidine to the molten top agar.

The *Allium* test was performed according to the procedure described by Babich et al. (1997). Uniform‐sized *Allium cepa* L. bulbs were purchased from local markets in Erzurum. The yellow–brown outer scales of the bulbs were peeled off. The bulbs were immersed into distilled water (negative control group), ethyl methanesulfonate (EMS) solution (positive control group) or test solutions at varying concentrations. After 48‐h incubation period at 25°C in the dark, the root tips were collected from the bulbs, fixed in 45% acetic acid–1% HCl (9:1), stained with 2% aceto‐orcein, and examined under a light microscope (400 ×) to calculate the mitotic index (MI) and identify chromosomal abnormalities. 5000 mitotic root cells from three independent experiments, each performed in duplicate, resulting in a total of six biological samples (*n* = 6) per test group, were scored and the results were expressed as mean percentage (%) values, and statistically analyzed Fisher's exact test and Linear trend (*p* < 0.05) (Feretti et al. 2007). The mitotic index (MI) was calculated according to the standard formula:

$$\mathrm{MI}=\left(\text{Number of dividing cells}/\text{Total number of cells}\right)\times 100$$

### 2.13 Morphological Study

The morphological analysis and identification were carried out using a Leica S8AP0 stereo microscope. A total of twenty specimens were examined to study the stems, leaves, flowers, and roots of *R. damascena*. Detailed descriptions and illustrations were provided for each part.

### 2.14 Quantitative Analysis of Secondary Metabolites

Chemical characterization of the most active extract was carried out using a targeted Liquid Chromatography–Tandem Mass Spectrometry (LC–MS/MS) approach based on a bioactivity‐guided selection strategy. The extract exhibiting the strongest biological activity was prioritized for detailed profiling, in line with validated Ultra‐High Performance Liquid Chromatography–Electrospray Ionization–Tandem Mass Spectrometry (UHPLC–ESI–MS/MS) methodologies reported in the literature (Can et al. 2024).

Analyses were performed at the Atatürk University Eastern Anatolia High Technology Application and Research Center (DAYTAM) using an Agilent 6460 Triple Quadrupole LC–MS/MS system coupled with an Agilent 1260 UHPLC unit. Chromatographic separation was achieved on an Agilent Poroshell 120 EC‐C18 column (4.6 × 100 mm, 3.5 μm). The column temperature was maintained at 30°C, with a flow rate of 0.40 mL/min and an injection volume of 5 μL. The total run time was 25 min.

The mobile phase consisted of (A) water containing 0.1% formic acid and (B) methanol containing 0.1% formic acid. A gradient elution program analogous to that described by Can et al. (2024) was employed to ensure optimal separation of the targeted phenolic compounds.

Ionization was carried out using electrospray ionization (ESI) in both positive and negative modes, depending on the chemical nature of the analytes. Data acquisition was performed in Multiple Reaction Monitoring (MRM) mode over a mass range of m/z 50–1300 using Agilent MassHunter software.

Compound identification and quantification were achieved based on retention time matching and MRM transitions, supported by comparison with authentic reference standards. External calibration curves were used for quantification, and method performance parameters, including limits of detection (LOD) and quantification (LOQ), were determined according to signal‐to‐noise criteria (3.3 and 10 σ/S, respectively), following validated UHPLC–ESI–MS/MS protocols (Can et al. 2024).

Sample preparation involved dissolving the extract in methanol, followed by dilution, filtration through a 0.22 μm membrane filter, and analysis in triplicate to ensure reproducibility. Quantitative results were expressed as ng/mL (mean ± standard deviation), and compounds below detection limits were reported as not detected (ND) or below the limit of quantification (LOQ).

### 2.15 Statistical Analysis

All experiments were performed in triplicate (*n* = 3), and results are expressed as mean ± standard deviation (SD). Due to the small sample size, statistical differences among groups were evaluated using the non‐parametric Kruskal–Wallis test. When a significant overall difference was detected (*p* < 0.05), Dunn's post hoc test with Bonferroni correction was applied for multiple pairwise comparisons. IC_50_ values were calculated from concentration–response curves using nonlinear regression analysis, and are reported as μg/mL. IC_50_ values were only determined for samples reaching ≥ 50% inhibition within the tested concentration range; otherwise, results are indicated as “not determined (ND)”. All statistical analyses were performed using SPSS software (IBM SPSS Statistics 20, IBM Corporation, Armonk, NY, USA).

## 3 Results

### 3.1 Extraction

Among the various plant parts analyzed, the highest extraction yield was obtained from the leaves, with a yield of 29.69%, indicating a higher concentration of extractable compounds in this part. In contrast, the branches exhibited the lowest extraction yield, with only 7.91%, suggesting a comparatively lower presence of soluble constituents. The yields of the obtained extracts were as follows: flowers yielded 14.15%, buds yielded 7.98%, branches (pruning residues) yielded 7.91%, and leaves (pruning residues) yielded 29.69%.

### 3.2 FTIR‐ATR Analysis

FTIR–ATR data were interpreted at the functional group level, while compound‐specific identification and quantification were supported by LC–MS/MS analysis to ensure consistency between spectroscopic observations and chemical composition.

**FIGURE 1:**
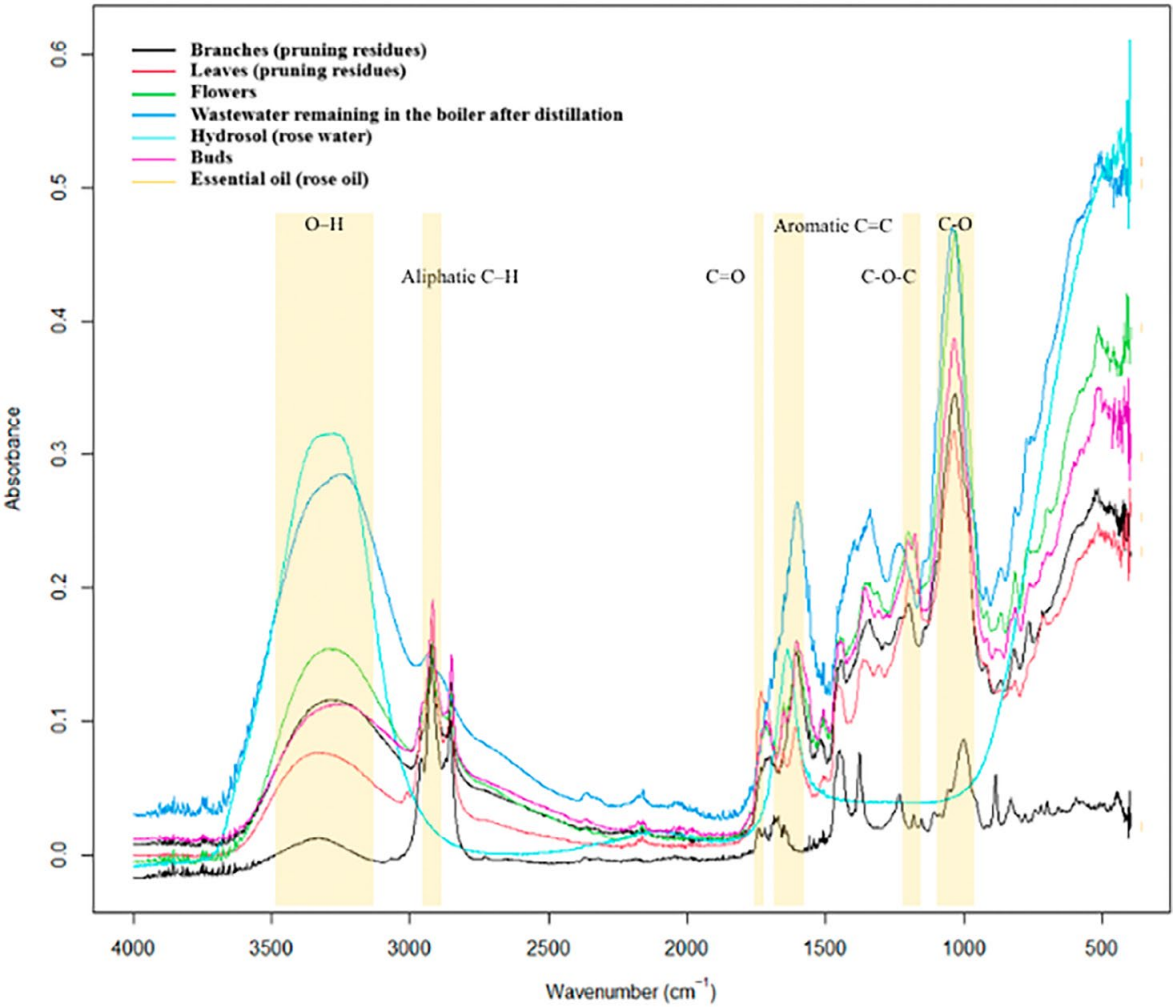
FTIR‐ATR spectra (4000–400 cm^−1^) of branches, leaves, flowers, wastewater after distillation, hydrosol, buds, and essential oil. Major bands were observed at ~3300 cm^−1^ (O–H), ~2920 cm^−1^ (C–H), ~1720 cm^−1^ (C=O), ~1620 cm^−1^ (C=C), and 1040–1220 cm^−1^ (C–O), indicating compositional differences among samples.

Baseline correction was applied to all spectra to eliminate background noise and enable clearer comparison of characteristic absorption bands. FTIR–ATR spectra of samples obtained from different plant parts and processing by‐products were recorded in the 400–4000 cm^−1^ range and are presented in Figure 1. The corrected spectra revealed distinct chemical profiles among the samples.

**FIGURE 2:**
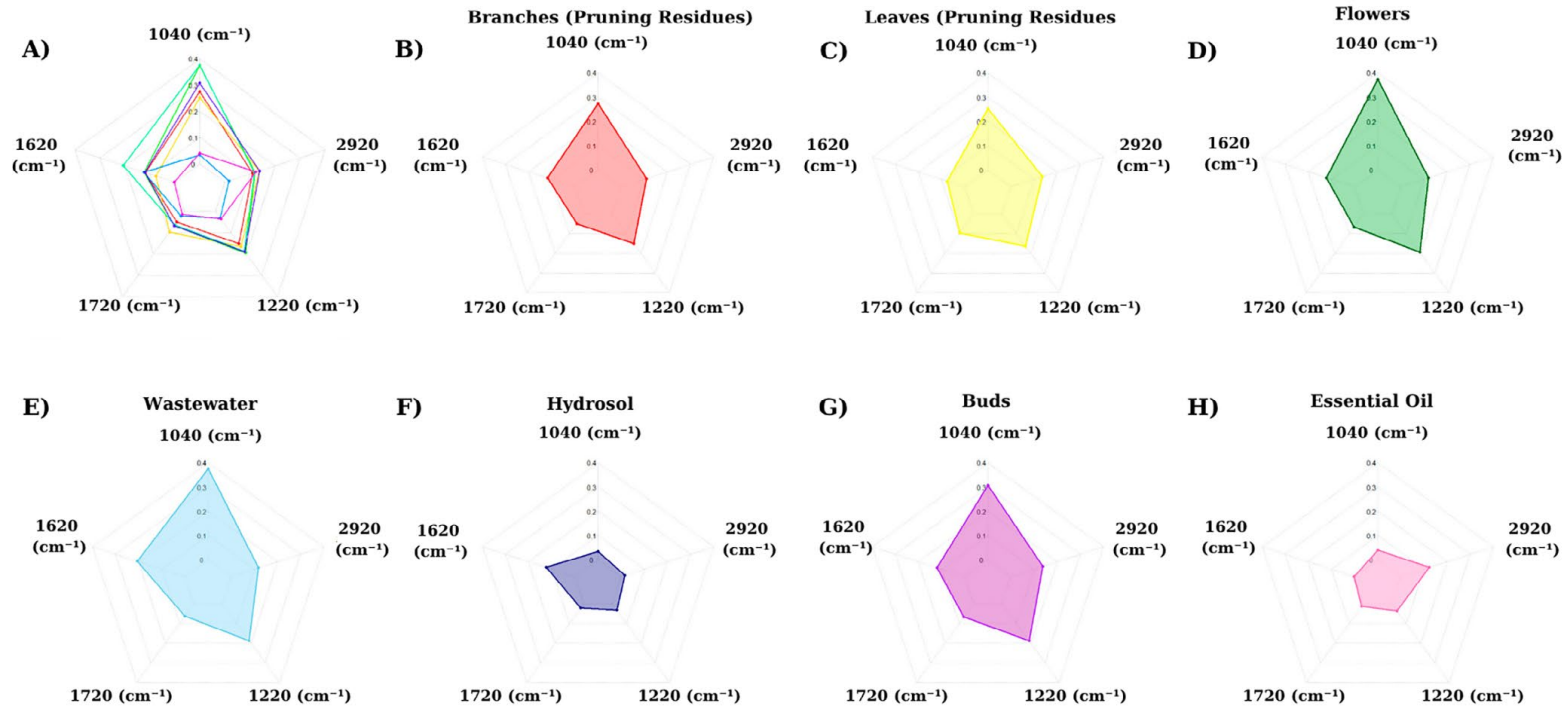
Radar charts representing the comparative FTIR‐ATR absorbance intensities of rose‐derived samples at 1040, 1220, 1620, 1720, and 2920 cm^−1^. (A) Overlay comparison of all samples. Individual profiles are shown for (B) branches (pruning residues), (C) leaves (pruning residues), (D) flowers, (E) wastewater remaining after distillation, (F) hydrosol, (G) buds, and (H) essential oil. The radial scale was fixed between 0 and 0.5 absorbance units to enable direct comparison of relative band intensities among samples.

Selected wavenumber regions, including 1000–1100 cm^−1^ (C–O), 1200–1250 cm^−1^ (C–O), 1600–1650 cm^−1^ (C=C), 1700–1750 cm^−1^ (C=O), and 2900–2950 cm^−1^ (C–H), were used to visualize compositional differences (Figure 2). These bands enabled differentiation of lignocellulosic structures, phenolic‐rich materials, and aliphatic components.

Woody tissues (branches and pruning residues) exhibited strong absorption bands in the 1000–1200 cm^−1^ region and characteristic signals in the 1500–1600 cm^−1^ range, indicating a lignocellulosic structure. A weak band around 1732 cm^−1^ was also observed.

Flower and bud samples showed prominent bands in the 1600–1650 cm^−1^ and 1700–1750 cm^−1^ regions, along with a broad O–H stretching band between 3000 and 3600 cm^−1^, indicating the presence of phenolic and oxygenated functional groups.

In processed samples such as distillation wastewater, strong bands were observed in the 1000–1200 cm^−1^ region, together with broad O–H absorption in the 3000–3600 cm^−1^ range.

The hydrosol spectrum was dominated by a broad O–H band (~3300 cm^−1^), accompanied by weak signals in the 1000–1100 cm^−1^ region.

Rose oil exhibited strong aliphatic C–H stretching bands in the 2800–3000 cm^−1^ region and a distinct carbonyl band in the 1700–1750 cm^−1^ range, while absorption in the 3200–3600 cm^−1^ region was negligible. Summary of key FTIR‐ATR band assignments for rose‐derived samples was given in Table 1.

**TABLE 1:** Summary of key FTIR‐ATR band assignments for rose‐derived samples.

Wavenumber (cm^−1^)	Assignment	Biochemical significance	Sample with highest intensity	References
~3300	O‐H stretching	Polysaccharides (cellulose, pectin), water, alcohols, phenols	Rose flower, rose bud, decoction pot waste	(Da Costa et al. 2020; Ribeiro et al. 2001)
3000–2800	C‐H stretching (CH_2_, CH_3_)	Aliphatic compounds (waxes, fatty acids, terpenes, lignin side chains)	Rose oil, rosewood pruning waste	(Da Costa et al. 2020)
~1732	C=O stretching	Carbonyl groups in hemicellulose (acetyl, uronic esters), pectin, and lignin‐carbohydrate complexes	Decoction pot waste, rose flower	(Smidt et al. 2008)
~1630	C=C aromatic ring stretching/amide I	Aromatic skeleton of lignin/proteins and peptides	Rosewood pruning waste (lignin), rose flower (protein)	(Da Costa et al. 2020; Smidt et al. 2008)
~1508	Aromatic skeletal vibrations	Lignin (specific marker)	Rosewood pruning waste (Branch & Knot)	(Faix 1991; Schwanninger et al. 2004)
1200–900	C‐O, C‐C, C‐O‐C stretching	Cellulose and hemicellulose (carbohydrate fingerprint region)	Rose flower, rose bud, decoction pot waste	(Da Costa et al. 2020)

All spectra were recorded in triplicate and are presented as representative profiles after preprocessing (baseline correction, normalization, and offsetting where necessary).

### 3.3 Enzyme Inhibition Assays

Table 2 presents the IC_50_ values and enzyme inhibitory activities of the tested samples. In the *α*‐glucosidase inhibition assay, flowers exhibited the strongest inhibition with the lowest IC_50_ value (37 μg/mL), followed by branches (73 μg/mL), buds (79 μg/mL), and leaves (223 μg/mL). Wastewater showed significantly weaker inhibition (IC_50_ = 811 μg/mL), while hydrosol and essential oil did not exhibit measurable activity. Acarbose, the reference inhibitor, had a much higher IC_50_ value (3248 μg/mL), indicating that the tested samples, particularly the flowers, were more potent inhibitors of *α*‐glucosidase.

**TABLE 2:** Antidiabetic and anticholinesterase activities of samples.

Samples	Antidiabetic activity assays		Anticholinesterase activity assays	
	*α*‐Glucosidase inhibitory activity (%) (at 500 μg/mL ± SD) [IC_50_ (μg/mL)]	*α*‐Amylase inhibitory activity (%) (at 5000 μg/mL ± SD)	AChE inhibitory activity (%) (at 100 μg/mL ± SD)	BChE inhibitory activity (%) (at 1000 μg/mL ± SD)
Flowers	97.10 ± 0.31 (IC_50_ = 37 μg/mL)	26.23 ± 3.49	14.39 ± 4.23	14.31 ± 2.57
Branches	97.77 ± 0.20 (IC_50_ = 73 μg/mL)	15.89 ± 2.91	12.30 ± 4.49	19.90 ± 3.97
Leaves	94.65 ± 0.20 (IC_50_ = 223 μg/mL)	34.28 ± 2.13	17.62 ± 0.60	20.35 ± 2.50
Buds	95.68 ± 0.41 (IC_50_ = 79 μg/mL)	23.44 ± 3.32	26.37 ± 4.65	11.02 ± 1.25
Wastewater	27.33 ± 2.11 (IC_50_ = 811 μg/mL)	32.47 ± 2.00	16.81 ± 3.75	17.58 ± 3.23
Hydrosol	ND	70.15 ± 2.54	15.72 ± 0.74	16.81 ± 1.00
Essential oil	ND	38.13 ± 4.25	20.70 ± 3.40	20.59 ± 2.72
Acarbose	4.96 ± 1.57 (IC_50_ = 3248 μg/mL)	74.61 ± 1.77	—	—
Donepezil	—	—	100 ± 1.21	99.12 ± 0.28

*Note:* Values are expressed as mean ± SD (*n* = 3). Statistical analysis was performed using Kruskal–Wallis test followed by Dunn's post hoc test (*p* < 0.05). IC_50_ values (μg/mL) were calculated by nonlinear regression.

Abbreviations: ND, not detected; SD, standard deviation.

At a fixed concentration (500 μg/mL), flowers (97.10%), branches (97.77%), and buds (95.68%) exhibited similarly high *α*‐glucosidase inhibition, with no significant difference among them (*p* < 0.05), whereas leaves (94.65%) showed slightly lower activity. In contrast, wastewater (27.33%) demonstrated significantly lower inhibition, and no activity was observed for hydrosol and essential oil. Acarbose showed significantly lower inhibition (4.96%) compared to all plant extracts.

Significant differences were also observed in *α*‐amylase inhibitory activity among the samples (*p* < 0.05). Hydrosol (70.15%) and acarbose (74.61%) exhibited the highest inhibition, followed by essential oil (38.13%), leaves (34.28%), and wastewater (32.47%), whereas branches (15.89%) showed the lowest activity.

For anticholinesterase activity, AChE inhibition varied significantly among samples (*p* < 0.05), with buds (26.37%) showing relatively higher activity compared to other plant parts, while the remaining samples exhibited moderate to low inhibition (12.30%–20.70%). BChE inhibition also differed significantly (*p* < 0.05), with leaves (20.35%) and branches (19.90%) showing higher activity compared to other samples. Donepezil, the reference inhibitor, demonstrated nearly complete inhibition of both AChE (100%) and BChE (99.12%). Hydrosol and essential oil did not exhibit *α*‐glucosidase inhibition, although their *α*‐amylase inhibition was moderate to high.

### 3.4 Antioxidant Capacity Assays

The antioxidant capacities of the samples analyzed in our study were evaluated via ABTS^•+^ and DPPH^•^ radical scavenging activities, and the results are presented in Table 3. Calibration curves created with standard substances were used to determine the concentrations at which the percent inhibition values of the samples could be compared most meaningfully.

**TABLE 3:** ABTS^•+^ and DPPH^•^ scavenging activity test results.

Samples	Antioxidant activity assays	
	ABTS^•+^ scavenging activity (% inhibition at 70 μg/mL ± SD)	DPPH^•^ scavenging activity (% inhibition at 100 μg/mL ± SD)
Flowers	90.838 ± 0.959	72.674 ± 0.665
Branches	95.030 ± 0.836	82.610 ± 0.163
Leaves	93.492 ± 0.677	79.265 ± 0.489
Buds	96.673 ± 0.541	72.678 ± 0.552
Wastewater	75.350 ± 0.416	71.000 ± 0.467
Hydrosol	ND	2.814 ± 3.967
Essential oil	ND	1.984 ± 1.143
*α*‐Tocopherol	19.569 ± 1.04	73.885 ± 7.69
Trolox	99.527 ± 0.32	93.008 ± 0.077

*Note:* Values are expressed as mean ± SD (*n* = 3). Statistical analysis was performed using Kruskal–Wallis test followed by Dunn's post hoc test (*p* < 0.05). IC_50_ values (μg/mL) were calculated by nonlinear regression.

Abbreviations: ND, not detected; SD, standard deviation.

Statistical analysis indicated that antioxidant activities differed significantly among the tested samples (*p* < 0.05). As a result of serial dilutions performed in ABTS^•+^ radical scavenging activity tests, it was determined that the most meaningful comparison could be made at a concentration of 70 μg/mL. At this concentration, the highest inhibition rate was observed in the buds sample (96.673%), followed by branches (95.030%) and leaves (93.492%), with no significant difference among these samples (*p* < 0.05). The flower sample also exhibited high activity (90.838%), although it was significantly lower than the top‐performing samples. In contrast, hydrosol and essential oil did not exhibit detectable ABTS^•+^ radical scavenging activity at this concentration.

When DPPH^•^ radical scavenging activity was examined at 100 μg/mL, branches (82.610%) and leaves (79.265%) exhibited the highest activities after the standards. Bud and flower samples showed similar activity (72.678% and 72.674%, respectively), with no significant difference between them (*p* < 0.05). In contrast, the wastewater sample showed relatively lower activity (71%), while hydrosol (2.814%) and essential oil (1.984%) exhibited significantly lower antioxidant effects compared to all other samples (*p* < 0.05).

The results of the CUPRAC and FRAP reducing activity assays are presented in Table 4. According to Table 4, the highest CUPRAC reducing activity was detected in the buds extract with values of 18.09 μmol TE/g and 36.86 μmol GAE/g. In contrast, the flowers extract exhibited the highest FRAP reducing activity, reaching 16.32 μmol TE/g and 2.37 μmol GAE/g. Among the tested samples, leaves and wastewater showed comparatively lower antioxidant reducing capacities, while hydrosol and essential oil samples demonstrated very limited or undetectable activity in some assays.

**TABLE 4:** CUPRAC and FRAP reducing activity test results.

Samples	CUPRAC reducing activity (μmol TE/g)	CUPRAC reducing activity (μmol GAE/g)	FRAP reducing activity (μmol TE/g)	FRAP reducing activity (μmol GAE/g)
Buds	18.09	36.86	13.24	2.20
Flowers	15.43	31.60	16.32	2.37
Branches	13.16	26.24	12.35	2.16
Wastewater	7.48	15.31	11.63	2.12
Leaves	6.26	12.63	8.97	1.98
Hydrosol	ND	ND	ND	1.50
Essential oil	ND	ND	0.25	1.51
Standards	Trolox	Gallic acid	Trolox	Gallic acid

*Note:* ND, not determined.

In general, parts of plant‐derived samples, especially branches, buds, and leaves, attracted attention with their strong antioxidant activity capacity; however, it was observed that hydrosol and essential oil samples exhibited quite limited effect in this respect. Comparative results of Trolox and *α*‐tocopherol reference substances reveal that the radical scavenging potential of plant samples can compete with synthetic antioxidants in some cases.

### 3.5 Determination of Antimicrobial Activity

The tested samples generally exhibited stronger antimicrobial activity against Gram‐positive bacteria and yeast compared to Gram‐negative bacteria. Among the investigated samples, flowers showed antimicrobial activity against *Staphylococcus aureus* ATCC 6538 with an MIC value of 625 μg/mL, while weaker activity was observed against *Pseudomonas aeruginosa* ATCC 9027 and *Candida albicans* ATCC 10231 (MIC values of 2500 and 1250 μg/mL, respectively). Buds, branches (pruning residues), and essential oil demonstrated notable antimicrobial activity against both *S. aureus* and *C. albicans* , each with MIC values of 625 μg/mL, and moderate activity against *P. aeruginosa* (MIC = 1250 μg/mL). Leaves (pruning residues) exhibited activity against *S. aureus* at 625 μg/mL and against *C. albicans* at 1250 μg/mL, whereas wastewater remaining in the boiler after distillation showed the highest activity against *C. albicans* with an MIC value of 625 μg/mL. Hydrosol displayed comparatively weak antimicrobial activity, with MIC values of ≥ 2500 μg/mL against *S. aureus* and *P. aeruginosa* , and 2500 μg/mL against *C. albicans* (Table 5).

**TABLE 5:** Minimum inhibitory concentration (MIC, μg/mL).

Extracts	*S. aureus*	*P. aeruginosa*	*C. albicans*
	ATCC 6538	ATCC 9027	ATCC 10231
Flowers	625	2500	1250
Buds	625	1250	625
Essential oil (rose oil)	625	1250	625
Hydrosol (rose water)	> 2500	> 2500	2500
Branches (pruning residues)	625	1250	625
Leaves (pruning residues)	625	2500	1250
Wastewater remaining in the boiler after distillation	1250	2500	625
Ampicillin	0.125 >	4	—
Ciprofloxacin	0.125 >	0.125 >	—
Fluconazole	—	—	32

### 3.6 Biosafety Assessment

The Ames/*Salmonella* assay results showed that *R. damascena* extracts and essential oil did not exhibit any genotoxic potential in *S. typhimurium* strains TA1535, TA1537, TA97a, TA98, and TA100 at all tested concentrations up to 500 μg/plate (Table 6). Similarly, no mutagenic activity was observed in the *E. coli* WP2uvrA assay under the same experimental conditions. The detailed Ames/*Salmonella* and *E. coli* WP2 mutagenicity results are provided in the Supporting Information (Tables S1–S6).

**TABLE 6:** The Ames/*Salmonella* and *E. coli* WP2 bacterial genotoxicity test results.

Test groups (μg/petri)	Number of revertant colonies of bacterial tester strains					
	(Mean ± SD)					
	*S. typhimurium*	*S. typhimurium*	*S. typhimurium*	*S. typhimurium*	*S. typhimurium*	*E. coli*
	TA1535	TA100	TA1537	TA97a	TA98	WP2
**Flowers**						
100	26.3 ± 2.34	48.2 ± 4.36	22.5 ± 2.95	42.7 ± 2.73	36.3 ± 3.67	44.0 ± 5.29
200	27.5 ± 3.72	48.5 ± 3.02	23.5 ± 3.27	38.8 ± 2.93	36.3 ± 2.66	44.8 ± 3.19
300	27.0 ± 2.37	49.8 ± 5.74	20.5 ± 0.84	41.0 ± 3.74	38.3 ± 3.14	46.8 ± 6.18
400	26.0 ± 2.53	49.2 ± 5.53	20.2 ± 2.04	42.5 ± 5.17	36.7 ± 2.58	47.3 ± 3.88
500	27.3 ± 2.42	48.2 ± 3.31	23.2 ± 2.64	42.3 ± 4.59	36.5 ± 3.94	43.5 ± 2.95
**Branches**						
100	25.3 ± 3.78	47.3 ± 3.72	22.5 ± 2.51	40.2 ± 4.96	39.0 ± 4.34	44.5 ± 3.39
200	28.5 ± 2.35	46.0 ± 4.86	23.3 ± 2.88	41.2 ± 2.71	36.8 ± 4.36	43.0 ± 5.66
300	28.3 ± 3.27	48.5 ± 5.89	22.8 ± 2.40	41.8 ± 3.60	38.5 ± 3.21	46.8 ± 2.56
400	27.5 ± 2.07	47.0 ± 3.03	22.0 ± 2.83	39.8 ± 4.62	36.0 ± 3.10	44.2 ± 5.12
500	25.8 ± 3.76	44.5 ± 3.83	23.3 ± 1.37	42.8 ± 3.13	36.5 ± 4.46	43.0 ± 5.33
**Leaves**						
100	27.7 ± 3.78	49.0 ± 4.29	22.3 ± 2.42	40.0 ± 4.90	35.3 ± 3.20	45.7 ± 4.89
200	27.0 ± 3.52	49.3 ± 3.78	24.0 ± 2.97	41.3 ± 6.44	36.2 ± 3.19	46.0 ± 4.56
300	24.8 ± 2.32	45.5 ± 3.73	21.8 ± 1.72	43.8 ± 3.06	35.7 ± 3.56	47.0 ± 5.90
400	27.2 ± 2.93	47.3 ± 6.77	23.3 ± 2.07	41.3 ± 5.61	38.8 ± 4.67	43.2 ± 5.53
500	27.0 ± 2.61	48.3 ± 4.46	22.8 ± 1.83	42.2 ± 4.40	37.7 ± 4.23	44.8 ± 5.91
**Buds**						
100	27.7 ± 2.73	46.3 ± 5.13	22.8 ± 2.40	43.8 ± 2.23	38.7 ± 3.98	43.3 ± 4.23
200	27.3 ± 2.16	47.7 ± 1.97	21.0 ± 2.00	40.7 ± 4.84	35.0 ± 3.35	46.3 ± 3.50
300	26.8 ± 2.64	45.8 ± 4.62	21.7 ± 3.14	40.2 ± 5.04	35.7 ± 2.42	41.2 ± 4.58
400	26.8 ± 2.86	46.5 ± 5.28	23.3 ± 2.66	41.3 ± 3.78	39.0 ± 2.83	46.5 ± 4.85
500	28.0 ± 2.19	45.3 ± 5.16	21.0 ± 3.10	43.3 ± 4.46	40.2 ± 2.32	40.0 ± 3.29
**Wastewater**						
100	26.5 ± 2.59	47.2 ± 3.92	24.5 ± 2.51	41.2 ± 3.43	36.0 ± 4.34	44.5 ± 4.04
200	24.7 ± 3.98	47.0 ± 5.59	21.3 ± 1.63	40.7 ± 4.41	38.3 ± 2.73	46.0 ± 4.98
300	28.0 ± 3.10	46.3 ± 4.37	22.3 ± 2.34	40.5 ± 4.37	38.3 ± 4.08	44.8 ± 4.31
400	27.3 ± 3.27	48.2 ± 5.08	22.5 ± 3.39	42.3 ± 5.05	36.2 ± 2.93	43.8 ± 4.02
500	25.2 ± 3.31	46.3 ± 3.78	24.0 ± 3.22	43.0 ± 3.29	37.3 ± 4.63	44.2 ± 3.82
**Hydrosol**						
100	25.7 ± 2.34	48.0 ± 4.00	23.8 ± 2.79	40.3 ± 3.93	35.3 ± 3.61	44.2 ± 4.67
200	26.8 ± 2.64	47.2 ± 5.15	22.7 ± 3.44	43.7 ± 3.14	32.3 ± 1.63	40.8 ± 2.48
300	25.3 ± 3.27	50.0 ± 4.82	22.2 ± 3.06	39.2 ± 4.67	39.0 ± 3.63	45.0 ± 3.10
400	25.8 ± 3.31	47.3 ± 5.57	22.3 ± 2.58	44.2 ± 2.79	37.5 ± 1.76	40.8 ± 5.46
500	25.5 ± 3.56	49.0 ± 4.73	23.5 ± 2.07	38.3 ± 3.93	38.5 ± 4.93	44.5 ± 4.59
**Essential oil**						
100	24.8 ± 2.64	47.8 ± 4.96	24.5 ± 2.88	41.5 ± 3.27	34.7 ± 2.50	43.5 ± 4.89
200	26.2 ± 2.48	47.7 ± 6.22	25.2 ± 1.33	39.0 ± 3.74	36.7 ± 2.25	45.8 ± 5.88
300	29.7 ± 1.51	47.0 ± 4.60	23.7 ± 3.01	39.7 ± 4.23	40.2 ± 1.83	42.8 ± 2.40
400	26.5 ± 3.27	52.2 ± 1.72	21.3 ± 2.50	41.7 ± 4.63	39.0 ± 2.28	44.3 ± 2.94
500	26.0 ± 2.83	49.2 ± 3.49	23.7 ± 2.73	39.5 ± 1.87	37.0 ± 4.77	44.8 ± 5.71
**Positive controls**						
NaN_3_ (5 μg)	456.5 ± 10.50	558.0 ± 15.74				
9‐AA (50 μg)			368.07 ± 7.34	494.2 ± 12.73		
4‐NPD (2.5 μg)					447.7 ± 10.65	
MNNG (1 μg)						597.5 ± 16.23
**Negative control**						
DMSO (100 μL)	26.5 ± 2.35	46.2 ± 4.62	24.8 ± 2.86	40.0 ± 2.83	37.3 ± 3.56	44.8 ± 5.34

**FIGURE 3:**
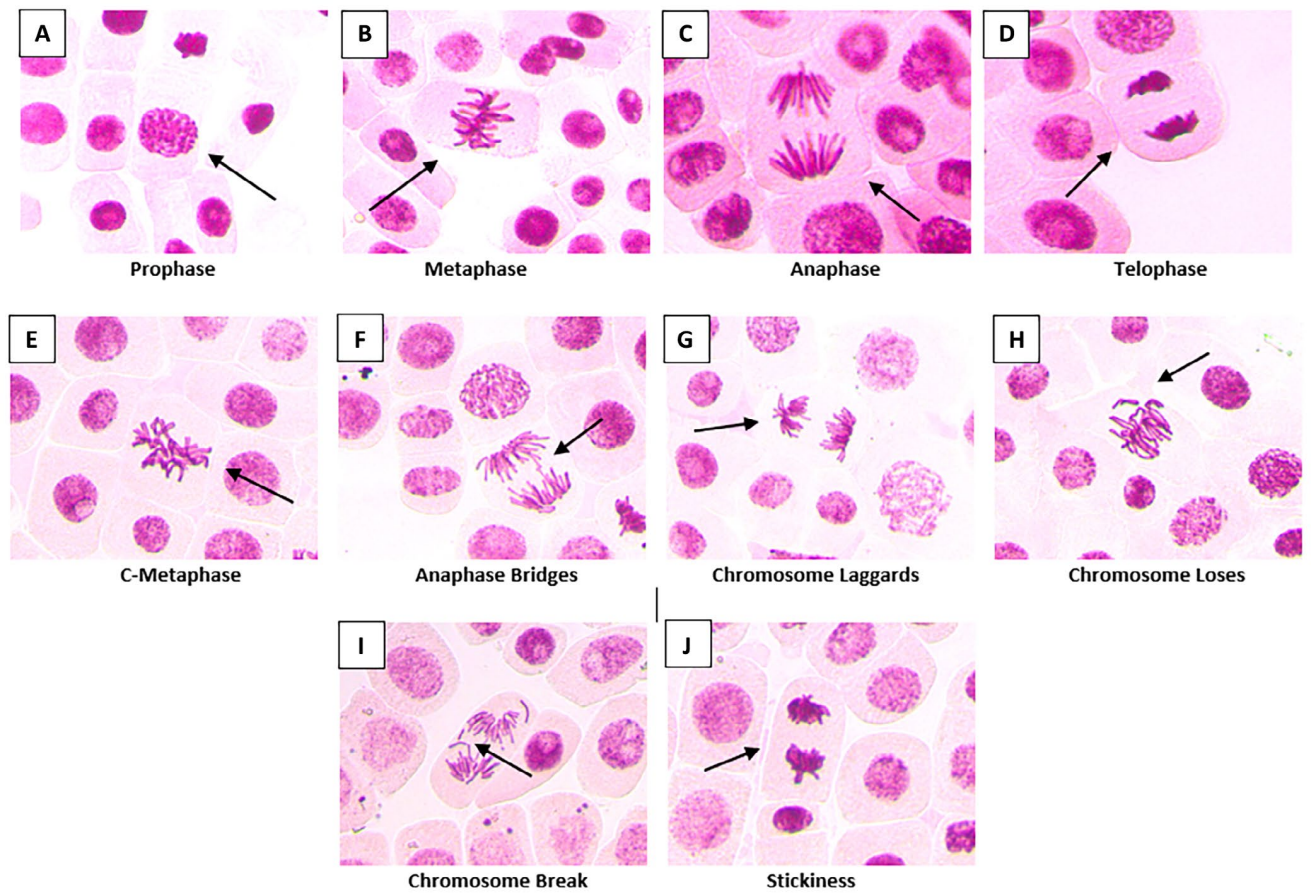
Typical stages of mitosis (A–D) and chromosomal aberrations (E–J) induced by EMS in mitotic *A. cepa* root cells.

In the *Allium cepa* assay, the positive control (EMS) significantly reduced the mitotic index (MI) and increased chromosomal aberrations at 25 mM. The observed aberrations included anaphase/telophase bridges, chromosome breakages, laggards, chromosome losses, C‐metaphases, disturbed ana‐telophases, and chromosomal stickiness (Figure 3).

In contrast, *R. damascena* extracts and essential oil did not induce significant chromosomal aberrations at any tested concentration up to 500 μg/mL. However, the flower extract caused a pronounced and dose‐dependent reduction in the mitotic index, decreasing MI values from 19.1% in the negative control to 12.0%, 11.8%, and 11.1% at increasing concentrations, respectively (Table 7). Although this reduction approached the level observed for the EMS positive control, it was not accompanied by a corresponding increase in chromosomal aberrations. Therefore, the observed effect is more indicative of a cytostatic influence on cell proliferation rather than a genotoxic or mutagenic response. The remaining extracts and essential oil did not significantly affect the mitotic index compared with the negative control.

**TABLE 7:** Mitotic index and chromosome aberration results of the *Allium* test.

Test groups (μg/mL)	Mitotic index (%)	Chromosome aberrations (%)							Statistical testing	
		AB	CB	CLag	CLos	CM	St	T	Fisher's exact test	Linear trend
**Flowers**										
100	12	0	0	0.1	0	0	0	0.1	NS	NS
200	11.9	0.1	0	0.1	0.1	0	0	0.3	NS	
300	11.7	0.1	0	0.1	0.1	0	0	0.3	NS	
400	11.4	0.1	0.1	0.2	0.1	0	0.1	0.6	NS	
500	11.1	0.2	0.1	0.2	0.2	0	0.1	0.8	NS	
**Branches**										
100	19.1	0.1	0	0	0	0	0	0.1	NS	NS
200	19.1	0.1	0	0	0.1	0	0	0.2	NS	
300	19.4	0.1	0	0.1	0.1	0	0.1	0.4	NS	
400	19.1	0.2	0.1	0.1	0.1	0	0.1	0.6	NS	
500	19.2	0.2	0	0.2	0.1	0	0.2	0.7	NS	
**Leaves**										
100	18.9	0.1	0.1	0.1	0.1	0	0	0.4	NS	NS
200	19	0.1	0.1	0.1	0	0	0	0.3	NS	
300	19.1	0.2	0.1	0.2	0.1	0	0.1	0.7	NS	
400	18.8	0.1	0.1	0.1	0.1	0	0.1	0.5	NS	
500	19.2	0.2	0	0.1	0.1	0	0.2	0.6	NS	
**Buds**										
100	19.1	0.1	0	0.1	0	0	0	0.2	NS	NS
200	19	0.1	0.1	0.1	0	0	0.1	0.4	NS	
300	19.2	0.1	0.1	0	0.1	0	0.1	0.4	NS	
400	19	0.2	0.1	0.1	0.2	0	0	0.6	NS	
500	18.7	0.3	0	0.1	0.1	0	0.1	0.6	NS	
**Wastewater**										
100	19	0.1	0.2	0.2	0.1	0	0	0.6	NS	NS
200	19	0.2	0	0.1	0	0	0.1	0.4	NS	
300	19.1	0.2	0.1	0.1	0.1	0	0.1	0.6	NS	
400	19.1	0.1	0.2	0	0.1	0	0.2	0.6	NS	
500	18.9	0.2	0.1	0.1	0.2	0	0	0.6	NS	
**Hydrosol**										
100	19.3	0.2	0	0.1	0.1	0	0.1	0.5	NS	NS
200	19.1	0.1	0.1	0.1	0.1	0	0.2	0.6	NS	
300	19.3	0.1	0	0	0.1	0	0	0.2	NS	
400	18.7	0.1	0.1	0.1	0	0	0.1	0.4	NS	
500	19	0.1	0.1	0.1	0.1	0.1	0.1	0.6	NS	
**Essential oil**										
100	19.1	0.2	0.2	0.1	0.1	0	0	0.6	NS	
200	19.1	0.2	0.1	0.1	0	0	0.1	0.5	NS	
300	19.1	0.1	0.1	0.1	0.1	0	0.1	0.5	NS	NS
400	19	0.3	0	0.2	0.1	0	0.2	0.8	NS	
500	18.9	0.3	0.1	0.1	0.2	0	0.1	0.8	NS	
**Control groups**										
Negative control: ddH_2_O	19.1	0.1	0.1	0.1	0	0	0.1	0.4		
Positive control: EMS (25 mM)	11.7	2.2	0.2	0.9	0.6	0.4	1.4	5.7		

Abbreviations: AB, anaphase bridge; CB, chromosome break; CLag, chromosome laggard; CLos, chromosome lose; CM, C‐metaphase; NS, not statistically significant compared to the negative control (*p* < 0.05); S, significant; St, stickiness; T, total rate of chromosome aberrations.

### 3.7 Morphological Study

**FIGURE 4:**
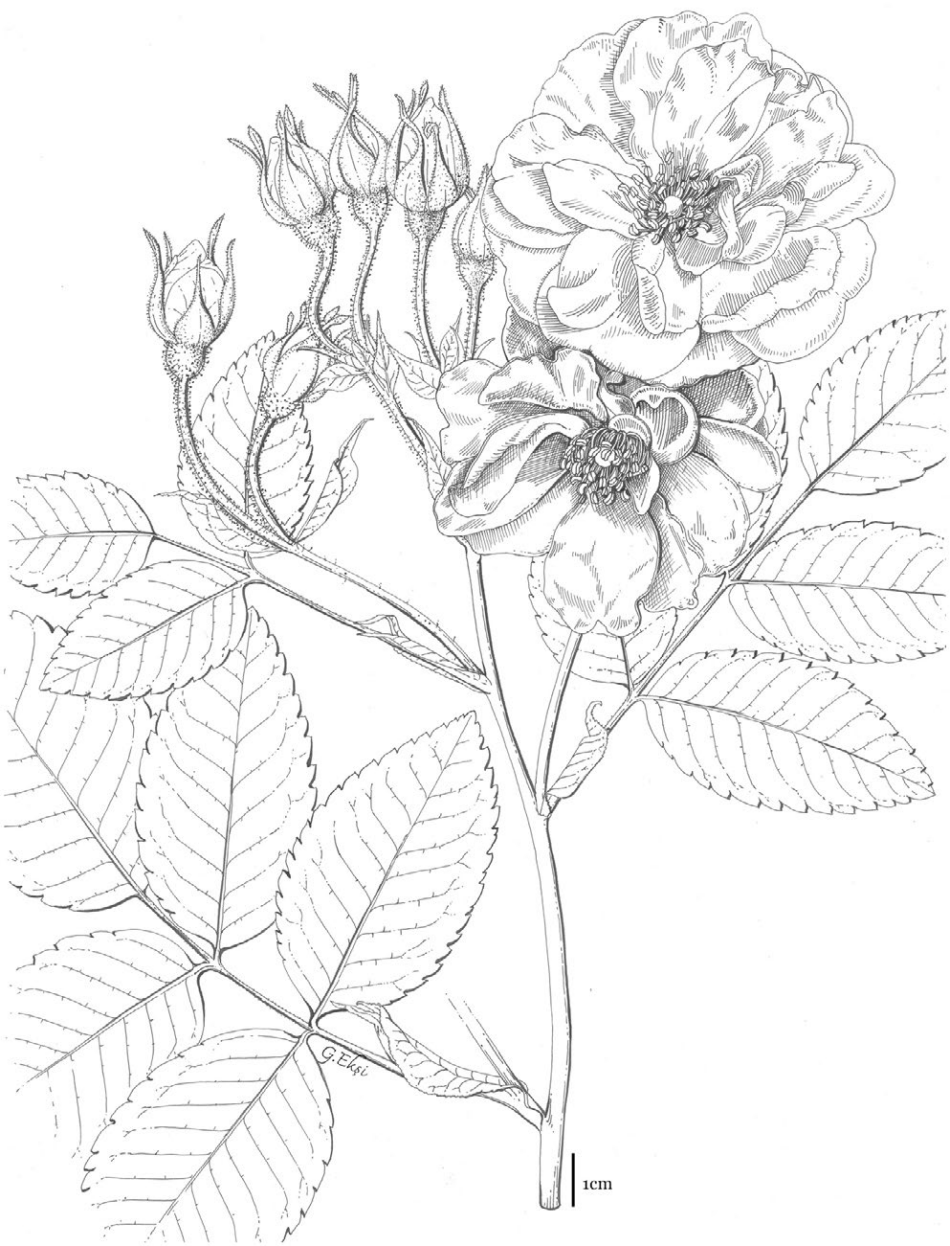
General appearance of *Rosa damascena* Mill. from Iğdır (Herb.no AUEF 1399). Drawn by Gülnur Ekşi Bona.

Plant shruby and deciduous. Stems up to 1–1.5 m and covered with prickles and bristles. Prickles 1–2 mm, dense on young shoots, deciduous as the stem aged. Stipules longer than half of petiole. Leaves 4–15 × 3–12 cm. Petioles 1–4 cm, glandular hairy and bristled. Leaflets 3–7 for per leaf, dark green. Inflorescence 1–7 flowered. Flowers pink, 4–8 layered; 4–10 cm in diameter. Pedicels 1–7 cm in length with 0.5–1.5 mm prickles. Hypanthium 4–7 × 3–6 mm, cup‐shaped, narrower at apex, glabrous at upper part. Receptacle densely covered with glandular hairs and prickles. Sepals 1–2 cm; entire or nearly entire; wide at the base; inner surface covered with lanate hairs, outer surface covered with glandular hairs. Petals pink; lined up in 5–8 layers. Stamens numerous and spirally arranged. Pistils numerous. Styles dense white pilose hairy. Stigma glabrous and wide (Figure 4).

### 3.8 Quantitative Analysis of Secondary Metabolites

A detailed quantitative analysis was performed on 35 different phenolic compounds, including gallic acid, ellagic acid, quinic acid, fumaric acid, pyrogallol, cyanidin‐3‐*O*‐glucoside, keracyanin chloride, chlorogenic acid, catechin, epicatechin, peonidin‐3‐*O*‐glucoside, 4‐hydroxybenzoic acid, epigallocatechin gallate, caffeic acid, syringic acid, vanillic acid, vitexin, naringin, hesperidin, *p*‐coumaric acid, sinapic acid, ferulic acid, taxifolin, rosmarinic acid, myricetin, vanillin, resveratrol, quercetin, luteolin, apigenin, naringenin, chrysin, isorhamnetin, galangin, and curcumin. These compounds were analyzed in various rose‐derived materials, including hydrosol (rose water), flowers, buds, pruning residues from leaves and branches, essential oil (rose oil), and the wastewater remaining in the boiler after distillation, using LC–MS/MS. Among these, 20 compounds were identified in the samples. Quinic acid was found to be the most abundant compound in the bud and flower extracts, with concentrations of 28,153.08 ng/mL and 22,661.91 ng/mL, respectively, with the highest *α*‐glucosidase, AChE inhibitor, and antioxidant activities. Additionally, high levels of gallic acid (14,893.14 ng/mL) and ellagic acid (10,636.74 ng/mL) were detected in the wastewater collected from the boiler after distillation. The detailed quantitative analysis of secondary metabolites in each sample is presented in Table 8.

**TABLE 8:** Quantitative analysis of secondary metabolites of samples.

Compounds	Wastewater remaining in the boiler after distillation	Branches (pruning residues)	Leaves (pruning residues)	Flowers	Hydrosol (rose water)	Buds	Essential oil (rose oil)
Quinic acid	19,139.0066	20,725.9828	9896.5189	22,661.9079	0.0000	28,153.0822	0.0000
Fumaric acid	0.0000	10.3990	72.8331	0.0000	0.0000	94.2099	0.0000
Gallic acid	14,893.1400	1383.0396	805.4934	1985.3669	0.0000	3132.2120	0.0000
Pyrogallol	0.0000	0.0000	0.0000	0.0000	0.0000	0.0000	0.0000
Keracyanin chloride	0.0000	0.0000	0.0000	0.0000	0.0000	0.0000	0.0000
Cyanidin‐3‐O‐glucoside	0.0000	0.0000	0.0000	0.0000	0.0000	0.0000	0.0000
Chlorogenic acid	61.4402	469.5558	3549.3520	11.5472	0.0000	55.7044	0.0000
Catechin	0.0000	8085.1729	0.0000	0.0000	22.131	0.0000	0.0000
Peonidin‐3‐O‐glucoside	13.538	0.0000	0.0000	30.894	0.0000	0.0000	0.0000
4‐OH‐benzoic acid	143.6572	0.0000	0.0000	0.0000	0.0000	0.0000	0.0000
Epicatechin	0.0000	0.0000	0.0000	0.0000	0.0000	62.1216	0.0000
Epigallocatechin gallate	0.0000	0.0000	0.0000	0.0000	0.0000	0.0000	0.0000
Caffeic acid	110.6293	0.0000	0.0000	49.8489	0.0000	0.0000	0.0000
Vanillic acid	0.0000	0.0000	0.0000	0.0000	0.0000	0.0000	0.0000
Vitexin	0.0000	0.0000	0.0000	0.0000	0.0000	0.0000	0.0000
Hesperidin	0.0000	0.0000	0.0000	0.0000	0.0000	0.0000	0.0000
Ellagic acid	10,636.7380	0.0000	0.0000	0.0000	920.7211	0.0000	647.5058
Naringin	0.0000	4863.4832	3119.3894	5931.1011	0.0000	8926.8017	0.0000
p‐Coumaric acid	0.0000	0.0000	0.0000	0.0000	0.0000	0.0000	0.0000
Ferulic acid	0.0000	0.0000	0.0000	0.0000	0.0000	0.0000	0.0000
Sinapic acid	0.0000	0.0000	0.0000	0.0000	501.3999	0.0000	0.0000
Rosmarinic acid	0.0000	0.0000	0.0000	0.0000	0.0000	0.0000	371.8938
Taxifolin	2366.4011	0.0000	0.0000	753.3125	0.0000	509.9969	0.0000
Syringic acid	0.0000	2059.3819	1047.3898	0.0000	0.0000	0.0000	0.0000
Vanillin	0.0000	0.0000	81.349	0.0000	0.0000	0.0000	0.0000
Myricetin	0.0000	0.0000	0.0000	0.0000	0.0000	0.0000	0.0000
Resveratrol	0.0000	0.0000	0.0000	0.0000	0.0000	0.0000	0.0000
Luteolin	0.0000	0.0000	0.0000	0.0000	0.0000	0.0000	0.0000
Quercetin	482.6984	242.9143	78.1517	555.4934	0.0000	558.0652	0.0000
Naringenin	0.0000	17.361	10.615	0.0000	0.1706	16.311	0.1536
Apigenin	17.384	0.0000	0.0000	13.931	0.0000	0.0000	0.0000
Isorhamnetin	0.0000	0.0000	0.0000	68.194	0.0000	108.1441	0.0000
Chrysin	0.0000	0.0000	0.0000	0.0000	0.0000	0.0000	0.0000
Galangin	0.0000	0.0000	0.0000	0.0000	0.0000	0.0000	0.0000
Curcumin	0.0000	0.0000	0.0000	0.0000	0.0000	0.0000	0.0000

## 4 Discussion

FTIR spectroscopy has been widely used as a rapid and reliable tool for the characterization of complex plant matrices and the discrimination of samples based on their chemical composition (Correia et al. 2016). The FTIR–ATR results revealed clear compositional differences among the various *R. damascena* plant parts and industrial by‐products, which can be directly linked to their distinct biochemical structures and functional roles. In particular, woody tissues such as branches and pruning residues exhibited spectral features characteristic of lignocellulosic biomass, including strong C–O stretching bands in the 1000–1200 cm^−1^ region and aromatic C=C vibrations in the 1500–1600 cm^−1^ range. These findings are consistent with the structural composition of plant cell walls, where cellulose, hemicellulose, and lignin constitute the dominant components (Faix 1991; Pandey 1999; Kacurakova et al. 2000; Liu et al. 2021).

In contrast, the spectra of flowers and buds were dominated by bands in the 1600–1650 cm^−1^ and 1700–1750 cm^−1^ regions, indicating a higher abundance of phenolic compounds and ester‐containing constituents. These functional groups are commonly associated with flavonoids, phenylpropanoids, and volatile compounds such as geraniol, citronellol, and their derivatives, which are known to be major components of *R. damascena* (Kumar et al. 2013; Abdullah et al. 2017). The comparatively lower intensity of ester‐related bands in bud samples suggests that the biosynthesis of volatile compounds may not yet be fully completed at this developmental stage, supporting previous observations on the maturation‐dependent accumulation of essential oil constituents (Baydar and Baydar 2005).

The processed by‐products, particularly distillation wastewater, exhibited strong C–O and O–H bands, reflecting the presence of polar constituents such as residual phenolics and polysaccharide‐derived compounds. This observation aligns with recent studies highlighting that significant amounts of bioactive phenolic compounds remain in rose processing residues and can be recovered for further applications (Dinkova et al. 2022; Kolev et al. 2025). These findings further support the concept of valorizing industrial by‐products as alternative sources of functional ingredients.

The hydrosol spectrum was dominated by O–H stretching vibrations, as expected for an aqueous system, but also displayed weak signals in the fingerprint region, indicating the presence of dissolved volatile compounds. Previous studies have similarly reported that hydrosols contain trace levels of aroma compounds transferred during distillation, contributing to their biological activity (Benoudjit et al. 2022; Tanjga et al. 2022).

Rose oil, on the other hand, showed a typical profile of non‐polar, lipid‐like constituents, characterized by strong aliphatic C–H stretching bands and a pronounced carbonyl signal. The absence of O–H stretching bands further confirms its hydrophobic nature and lack of polar constituents. Such spectral features are in agreement with the well‐documented composition of essential oils, which are dominated by terpenoid and ester compounds (Schulz et al. 2005).

The combined application of FTIR spectroscopy and LC–MS/MS analysis has become a valuable approach for comprehensive characterization of complex biological systems, providing complementary structural and chemical information that cannot be obtained by a single analytical technique alone (Keskin et al. 2024; Köktürk et al. 2022; Wanaragthai et al. 2025).

Overall, the FTIR–ATR findings demonstrate that different plant organs and industrial residues of *R. damascena* possess distinct chemical signatures that reflect their compositional diversity. When interpreted together with LC–MS/MS data, these results provide a comprehensive understanding of the phytochemical distribution within the plant and its by‐products. Importantly, the presence of phenolic and oxygenated functional groups in both plant materials and processing residues supports their observed antioxidant and enzyme inhibitory activities, highlighting their potential for sustainable utilization in functional food and nutraceutical applications.

Enzyme inhibition plays a crucial role in the management of both DM and AD as key metabolic and neurological enzymes are involved in disease progression. Targeting *α*‐glucosidase and *α*‐amylase can help regulate blood glucose levels in DM, while AChE and BChE inhibition is central to AD treatment by enhancing cholinergic transmission.

A study evaluated the *α*‐glucosidase inhibitory and blood glucose‐lowering effects of a methanol extract of *R. damascena* flowers (MRD) in normal and diabetic rats, compared to acarbose. Kinetic analysis showed MRD exerted strong, noncompetitive *α*‐glucosidase inhibition (98%), significantly higher than acarbose (51%, *p* < 0.0001) as in our study. Oral administration of MRD (100–1000 mg/kg) dose‐dependently reduced postprandial blood glucose levels in both normal and diabetic rats, significantly suppressing glucose spikes after maltose intake (*p* < 0.0001). The effect was comparable to acarbose (Gholamhoseinian and Fallah 2009).

Another study investigated the antidiabetic potential of volatile organic compounds (VOCs) extracted from the roots of *R. damascena* through hydrodistillation. The inhibitory effects on *α*‐amylase and *α*‐glucosidase were assessed, with IC_50_ values of 0.95 ± 0.20 mg/mL and 0.92 ± 0.11 mg/mL, respectively (Sharma et al. 2024). In our study, the essential oil obtained from rose petals exhibited no inhibitory effect on *α*‐glucosidase. Regarding *α*‐amylase inhibition, it exhibited approximately half the activity of acarbose.

In a study, it was investigated the hypoglycemic potential of *R. damascena* petal extracts. Aqueous and methanolic extracts were prepared, with total phenolic contents of 29.23 g GAE/L and 58.8 g GAE/L, respectively. In vitro *α*‐amylase inhibition assays demonstrated dose‐dependent effects, with inhibition ranging from 33.66% to 89.96% for the aqueous extract, 41.57% to 92.16% for the methanolic extract, and 23.4% to 81.82% for acarbose. Both extracts exhibited higher *α*‐amylase inhibitory activity than acarbose. In vivo studies in normal and alloxan‐induced diabetic mice showed that oral administration of the methanolic extract significantly reduced postprandial blood glucose levels after maltose loading in a dose‐dependent manner (Alsalti et al. 2022).

A study evaluated the anticholinesterase activity of *R. damascena* essential oil and aromatic waters using in vitro and *in silico* methods. The essential oil exhibited notable inhibition against AChE (60.86% ± 1.99%) and BChE (51.08% ± 1.70%) at 1000 μg/mL. Among the main components, phenylethyl alcohol showed the highest cholinesterase inhibition and was further analyzed via molecular docking (Senol et al. 2013).

In a study, the in vitro AChE inhibitory activities of hydroalcoholic extracts from five *R. damascena* cultivars (Bulgarica, Faik, Iranica, Complex‐635, and Complex‐637) grown in Isparta, Turkey were evaluated. All cultivars demonstrated significant AChE inhibitory activity. Among them, Complex‐635 exhibited the highest AChE inhibition with an IC_50_ value of 3.92 μg/mL, showing greater potency than other cultivars but lower than the reference drug donepezil (IC_50_ = 4.49 × 10^−3^ μg/mL) (Tarbiat et al. 2020).

Another study investigated the protective effects of aqueous *R. damascena* extract (DRE) against aluminum chloride (AlCl_3_)‐induced oxidative damage in an AD model of Wistar rats. Seven groups (*n* = 10) were studied, including control, sham, AlCl_3_‐treated, extract‐only (500 and 1000 mg/kg), and treatment groups receiving both AlCl_3_ (100 mg/kg) and DRE (500 and 1000 mg/kg). Behavioral tests showed that AlCl_3_ impaired spatial memory and significantly increased the time needed to find the hidden platform. Biochemical analysis revealed that AlCl_3_ increased AChE activity from 1.176 ± 0.173 to 3.62 ± 0.348, while 1000 mg/kg DRE reduced it to 1.56 ± 0.303. DRE also enhanced catalase and glutathione levels, reduced malondialdehyde (MDA) levels, and regulated AChE activity (Beigom Hejaziyan et al. 2023).

Our study evaluated the enzyme inhibition activities of various *R. damascena* samples, revealing that flowers exhibited the strongest *α*‐glucosidase inhibition, followed by buds, branches, and leaves, with wastewater showing the weakest effect. The flowers also showed significant anticholinesterase activity, particularly in inhibiting AChE. These results align with previous studies, such as Gholamhoseinian and Fallah (2009), which demonstrated strong *α*‐glucosidase inhibition and blood glucose‐lowering effects in rats (Gholamhoseinian and Fallah 2009). Similarly, Sharma et al. (2024) found potent *α*‐amylase and *α*‐glucosidase inhibition in volatile compounds from *R. damascena* roots. Furthermore, anticholinesterase activity in *R. damascena* essential oil, as reported by Senol et al. (2013), is consistent with our findings. Studies on *R. damascena* in AD's models also support its potential in reducing AChE activity and oxidative stress (Beigom Hejaziyan et al. 2023). Overall, our findings confirm *R. damascena* as a promising candidate for managing DM and AD.

Oxidative stress is a key factor in the development of various chronic diseases, including DM and AD. Antioxidants help counteract oxidative damage by neutralizing free radicals, thereby protecting cells from dysfunction and degeneration. Evaluating the antioxidant capacity of plant extracts provides valuable insight into their potential therapeutic benefits.

In a study comparing the antioxidant capacities of the essential oil obtained by hydrodistillation of fresh leaves of *R. damascena* and the 80% methanolic extract prepared from the leaves, the DPPH method was used for the measurement of free radical scavenging activity and the ferric ammonium thiocyanate (FTC) method was used for the evaluation of lipid peroxidation properties. According to the obtained data, it was observed that the extract had strong free radical scavenging activity compared to the standards (*p* < 0.03 IC_50_ of extract 2.24 μL, IC_50_ of BHT 110.98 μL). It was observed that the essential oil showed better antioxidant activity than the standards but weaker antioxidant activity than the extract (IC_50_ 3.54 μL) (Yassa et al. 2009).

In a study evaluating the antioxidant activities of *Rosa damascena* flowers before and after rose water production, total phenolic contents were found to be 276,022.93 mg gallic acid equivalent (GAE)/g in FF (fresh flower) extract and 248,972.96 mg GAE/g in SF (spent flower) extract. FF and SF extracts showed 74,511.65% and 75,941.72% antiradical activity at 100 ppm. The antioxidant activity of FF extract (372,260.96 mg/g) was higher than that of SF extract (351,360.84 mg/g) (Özkan et al. 2004).

In our studies, it was observed that antioxidant capacity was significantly high especially in plant extracts, while this effect decreased in processed rose products. This is probably related to the loss or oxidation of phenolic compounds that occur during processing. The findings seem to be consistent with the results reported in the literature. For example, there were studies showing that the phenolic content and antioxidant activity of extracts obtained from fresh *Rosa damascena* flowers were higher compared to processed products (Baydar and Baydar 2013).

The majority of *R. damascena* activity studies in the literature focus on essential oil. In a study, the main *R. damascena* compounds with antibacterial properties include essential oil, hydrosol, and absolute. Strong antibacterial action was exhibited by *R. damascena* essential oil and absolute against strains of *Escherichia coli, Pseudomonas aeruginosa, Carotovora, Staphylococcus aureus, Chromobacterium violaceum, and Erwinia*. The two bacteria most susceptible to *R. damascena*'s absolute essential oil were *E. coli and C. violaceum*. Both Gram‐positive and Gram‐negative bacteria are susceptible to the antibacterial properties of the absolute rose (Ulusoy et al. 2009).

Rose oil and several petal extracts were tested for minimum inhibitory concentrations (MICs) against acid‐fast, Gram‐positive, and two non‐Enterobacteriaceae Gram‐negative bacteria.

Gram‐positive bacteria, such as *S. aureus, B. subtilis, and Streptococcus pyogenes*, had MICs and MBCs of 0.125–2 mg/mL and 0.5–4, respectively, and were more sensitive than Gram‐negative bacteria. However, the most potent antifungal extract of petals was rose oil, while the least effective was the aqueous extract. The activity of the concrete against the investigated fungus was essentially the same as that of other fractionated extracts (Shohayeb et al. 2014).

In one study, the effects of rose water on the growth of methicillin‐resistant *Staphylococcus aureus* (MRSA) and *Candida albicans* were examined. At a concentration of about 2.2%, rose water inhibited the mycelial development of *Candida albicans* and decreased MRSA viability at 1 h (Maruyama et al. 2017).

In a reported study, the antibacterial activity of *R. damascena* oil was assessed against a wide range of microorganisms, including *Staphylococcus aureus* ATCC 25923, *Escherichia coli* ATCC 8739 (MIC = 1 μg/mL), and *Candida albicans* ATCC 10231 (MIC = 0.5 μg/mL) (Mahboubi et al. 2011).

The essential oils of *R. damascena* had a MIC value of 62.50 μg/mL and showed efficacy against Gram‐negative *Pseudomonas aeruginosa*. The oil of *Rosa* × *damascena* from the Javinan area had the highest inhibitory and antifungal properties against *Candida albicans* ATCC 10231 at MIC = 62.50 μg/mL. When *R*. × *damascena* essential oil was used to treat *S. aureus* ATCC 29737, the MIC value was found at 500 μg/mL (Ghavam et al. 2021). In our study, essential oil of *R. damascena* showed antimicrobial activity against *S. aureus* and *C. albicans* at MIC = 625 μg/mL.

Short‐term bacterial reverse mutation test systems have been widely used for genotoxic assessment of various synthetic compounds and natural components due to their high reliability, reproducibility, and low cost for decades (Hayashi 2022). In this regard, the Ames/*Salmonella* assay, employing a range of histidine (His^−^) auxotroph *S. typhimurium* tester strains carrying unique gene mutations in their His operon, has been one of the most widely used test systems in the genotoxic safety research for decades (Aydın et al. 2023; Karakaya et al. 2023; Mortelmans and Zeiger 2000).

The absence of mutagenic effects in both the Ames/*Salmonella* and *E. coli* WP2uvrA assays indicates that *R. damascena* extracts and essential oil do not induce gene mutations under the tested conditions. The use of multiple *S. typhimurium* strains targeting both base substitution (TA1535, TA100) and frameshift mutations (TA1537, TA97a, TA98) strengthens the reliability of this finding, as it demonstrates the lack of genotoxicity across different mutation mechanisms.

Furthermore, the *E. coli* WP2uvrA assay, which is particularly sensitive to certain mutagens not always detected by Salmonella strains, confirmed the absence of mutagenic activity, supporting the robustness of the safety assessment (Mortelmans and Riccio 2000).

The *Allium cepa* assay results further corroborate these findings at the chromosomal level, as no significant increase in aberrations was observed in treated groups compared to the control. The lack of chromosomal damage, together with the stable mitotic index, suggests that the tested samples do not exert cytotoxic or genotoxic effects under the experimental conditions.

The slight dose‐dependent reduction in mitotic activity observed for the flower extract may indicate a mild cytostatic effect rather than genotoxicity, as it was not accompanied by increased chromosomal abnormalities.

Overall, the combined results from bacterial and plant‐based assays provide strong evidence supporting the genotoxic safety of *R. damascena* extracts and essential oil, reinforcing their potential for use in functional and nutraceutical applications.

In cytogenotoxic safety perspective, the *Allium* test, one of the widely accepted cytogenotoxicity test systems in the literature (Ciğerci et al. 2023; Feretti et al. 2007; Leme and Marin‐Morales 2009).

The results confirm that all tested extracts and essential oil of *R. damascena* are safe at the tested concentrations in terms of cytogenotoxic potential. In addition, the potential of the flower extract to reduce cell proliferation without causing chromosomal aberration is important for future drug research.

Quinic acid, found in various medicinal plants, has been shown in studies to possess antioxidant, antidiabetic, anticancer, antimicrobial, antiviral, anti‐aging, and analgesic properties. Quinic acid, a natural polyphenol, effectively inhibits *α*‐glucosidase and anticholinesterase (Benali et al. 2024; Han et al. 2024; Orhan et al. 2007). Gallic acid and ellagic acid have been reported to exhibit AChE, *α*‐amylase, and *α*‐glucosidase inhibitory properties (Imededdine et al. 2023; Li et al. 2024; Oboh et al. 2016; Oh et al. 2021; Yin et al. 2018).

The observed differences in bioactivity among *R. damascena* plant parts can be attributed to their distinct phytochemical compositions and physiological roles. Floral tissues, which are metabolically active and involved in the reproduction and attraction of pollinators, are known to accumulate higher levels of secondary metabolites such as phenolic compounds, flavonoids, and volatile constituents. These compounds are strongly associated with antioxidant and enzyme inhibitory activities, which explains the pronounced bioactivity observed in flower extracts.

In contrast, woody tissues such as branches primarily serve structural functions and are therefore dominated by lignocellulosic components, including cellulose, hemicellulose, and lignin. These macromolecules have limited direct contribution to radical scavenging or enzyme inhibition, which may account for the comparatively lower bioactivity observed in branch extracts. However, the presence of residual phenolic compounds in these tissues may still contribute to moderate activity levels.

Similarly, buds represent an intermediate developmental stage, where biosynthesis of secondary metabolites is active but not yet fully completed, resulting in a phytochemical profile that can support significant, though variable, bioactivity. Leaves, on the other hand, function as primary sites of photosynthesis and defense, often accumulating phenolic compounds as protective agents against oxidative stress and environmental factors, which may explain their relatively high antioxidant and enzyme inhibitory potential.

Furthermore, the limited activity observed in hydrosol and essential oil samples may be related to their chemical composition. Hydrosols contain predominantly water‐soluble volatile fractions at low concentrations, while essential oils are largely composed of non‐polar terpenoids, which are less effective in assays based on electron or hydrogen donation compared to phenolic‐rich extracts.

Overall, these findings highlight that the differential bioactivity of *R. damascena* plant parts is closely linked to their biochemical roles and corresponding phytochemical profiles, emphasizing the importance of selecting appropriate plant matrices for targeted functional applications.

## 5 Conclusion

This study provides a comprehensive chemical, biological, and biosafety evaluation of different organs of *R. damascena* together with industrial processing residues, including hydrosol, essential oil, and distillation wastewater. Significant differences in extraction yield and chemical composition were observed among plant parts. FTIR‐ATR analysis distinguished lignocellulosic woody tissues from phenolic‐ and ester‐rich floral materials, while LC–MS/MS profiling identified 20 phenolic compounds, with quinic acid being predominant in flowers and buds. Notably, the detection of gallic and ellagic acids in distillation wastewater highlights the bioactive potential of this industrial by‐product. Biological assays demonstrated strong *α*‐glucosidase inhibition, particularly in flower extracts, and considerable antioxidant activity in buds, branches, and leaves, whereas hydrosol and essential oil showed limited effects under the tested conditions. Moderate antimicrobial activity was observed mainly against Gram‐positive bacteria and yeast. Genotoxicity assessments using Ames/*Salmonella*, *E. coli* WP2, and *Allium cepa* assays indicated no mutagenic or chromosomal damage at the tested concentrations.

Overall, the results suggest that *R. damascena* plant parts and processing residues contain bioactive compounds with potential functional relevance. However, further studies, including in vivo evaluation and formulation‐based investigations, are required to confirm their applicability in functional or industrial contexts.

## Author Contributions

**Burak Bayrak:** conceptualization, investigation, writing – original draft, methodology, data curation. **Satuk Buğra Alkuyruk:** conceptualization, investigation, methodology, data curation. **Hafize Yuca:** conceptualization, investigation, writing – original draft, funding acquisition, data curation, methodology, resources. **Yasemin Beyza Budak:** conceptualization, methodology, investigation, data curation. **Mehmet Karadayı:** conceptualization, investigation, writing – original draft, methodology, data curation. **Yusuf Gülşahin:** conceptualization, investigation, methodology, data curation. **Songul Karakaya:** conceptualization, investigation, writing – original draft, supervision, data curation, writing – review and editing, visualization. **Gülnur Ekşi Bona:** conceptualization, investigation, writing – original draft, methodology, data curation. **Alptuğ Atila:** conceptualization, investigation, methodology, data curation. **Elif Beyza Özer:** conceptualization, investigation, methodology, data curation. **Bilge Aydın:** conceptualization, investigation, writing – original draft, methodology, data curation. **Gamze Göger:** conceptualization, investigation, writing – original draft, methodology, data curation.

## Funding

We would like to express our gratitude to the Scientific and Technological Research Council of Turkey (TUBITAK) for supporting this research under the 2209‐A University Students Research Projects Support Program, with the project number 1919B012208044.

## Disclosure

The authors have nothing to report.

## Ethics Statement

The authors have nothing to report.

## Conflicts of Interest

The authors declare no conflicts of interest.

## Supporting information


**Table S1:** Mutagenicity results of *R. damascena* extracts, essential oil, hydrosol, and distillation wastewater in *S. typhimurium* TA1535 strain determined by the Ames bacterial reverse mutation assay.
**Table S2:** Mutagenicity results of *R. damascena* extracts, essential oil, hydrosol, and distillation wastewater in *S. typhimurium* TA100 strain determined by the Ames bacterial reverse mutation assay.
**Table S3:** Mutagenicity results of *R. damascena* extracts, essential oil, hydrosol, and distillation wastewater in *S. typhimurium* TA1537 strain determined by the Ames bacterial reverse mutation assay.
**Table S4:** Mutagenicity results of *R. damascena* extracts, essential oil, hydrosol, and distillation wastewater in *S. typhimurium* TA97a strain determined by the Ames bacterial reverse mutation assay.
**Table S5:** Mutagenicity results of *R. damascena* extracts, essential oil, hydrosol, and distillation wastewater in *S. typhimurium* TA98 strain determined by the Ames bacterial reverse mutation assay.
**Table S6:** Mutagenicity results of *R. damascena* extracts, essential oil, hydrosol, and distillation wastewater in *E. coli* WP2uvrA strain determined by the bacterial reverse mutation assay.

## Data Availability

The datasets used and/or analyzed during the current study are available from the corresponding author on reasonable request.
